# Challenging case of Familial Mediterranean Fever and its management

**DOI:** 10.1186/1546-0096-13-S1-P132

**Published:** 2015-09-28

**Authors:** A Al Hosani, ES Sharif

**Affiliations:** 1 Tawam Hospital, Paediatric, Abu Dhabi, United Arab Emirates; 2 Child Health Institute, Paediatric, Al Ain, Abu Dhabi, United Arab Emirates

## Background

Familial Mediterranean Fever (FMF) is an autosomal recessive disease which is characterized by recurrent, self-limiting, short attacks of serositis (peritonitis, pleuritis or arthritis), fever and erysipelas-like skin lesions along with a marked increase in acute phase reactants. Although FMF is not associated with immune-mediated tissue damage, however these individuals are prone to develop type AA amyloidosis and associated renal impairment progressing eventually to renal failure as the most severe long term complications. Colchicine is the best treatment option for the time being, however 10 to 15% of patient with FMF are unresponsive or intolerant to colchicine. for those cases recent literature review have shown successful use of biologic agents in management.

## The case

We present a challenging case of FMF in a 14 yrs old syrian girl who was diagnosed to have FMF at age of 6 years, she was started on colchicine at the age of 8 years, family history was significant for FMF and two of the family members developed renal amyloidosis requiering renal transplant. She presented to the clinic at 12 yrs old with bilateral ankle swelling. On examination she was found to have bilateral lower limb edema, her lab tests were significant for albumin of 18, urinalysis was positive for +4 proteins, renal biopsy was done which confirmed renal amyloidosis. The girl was on colchicine, we maximised the dose but the proteinuria persisted, she was also started on multiple courses of steroids and methotrexate with short lived improvement only. After reviewing the literature we found case reports that describe the successful use of biological agents especially anakinra (interleukin 1 receptor antagonist) in treatment of resistant cases of FMF with success. It was decided then to place her on anakinra, 2 months after being on anakinra we witnessed a significant improvement the edema totally resolved and amyloid protein has decreased from 203 to less than 5 (normal value).

## Discussion

The major cause of mortality in FMF is the insidious development of secondary (AA) amyloidosis with eventual renal failure. Treatment of FMF is centered on prevention of painful attacks and the development of amyloidosis. Colchicine has been the main stay for treatment of FMF since 1974. Since then colchicine has been used to prevent and treat the acute attacks of FMF but in 10-15 % of pt, the response to colchicine may be minimal or absent. These patients also have an increased risk of developing amyloidosis. For such cases some of the newly discovered biological agent like: anakinra (IL-1 receptor antagonist) and etanercept have been tried with promising results, however the the clinical efficacy and safety of these agents are yet to be determined

## Conclusion

The new discovery of biological agents has changes the outcome of many rheumatological diseases especially in paediatric population. A few colchicine resistant FMF patients have shown responsiveness to anakinra (an interleukin-1 receptor antagonist). However, the true efficacy and safety of treatment with biological agents remains uncertain because of the paucity of reports and absence of controlled trials.

## Consent to publish

Written informated consent for publication of their clinical details was obtained from the patient/parent/guardian/relative of the patient.

